# Activity of right premotor-parietal regions dependent upon imagined force level: an fMRI study

**DOI:** 10.3389/fnhum.2014.00810

**Published:** 2014-10-08

**Authors:** Nobuaki Mizuguchi, Hiroki Nakata, Kazuyuki Kanosue

**Affiliations:** Faculty of Sport Sciences, Waseda UniversityTokorozawa, Saitama, Japan

**Keywords:** motor imagery, premotor cortex, parietal cortex, grasp

## Abstract

In this study, we utilized functional magnetic resonance imaging (fMRI) to measure blood oxygenation level-dependent (BOLD) signals. This allowed us to evaluate the relationship between brain activity and imagined force level. Subjects performed motor imagery of repetitive right hand grasping with three different levels of contractile force; 10%, 30%, and 60% of their maximum voluntary contraction (MVC). We observed a common activation among each condition in the following brain regions; the dorsolateral prefrontal cortex (DLPFC), ventrolateral prefrontal cortex (VLPFC), supplementary motor area (SMA), premotor area (PM), insula, and inferior parietal lobule (IPL). In addition, the BOLD signal changes were significantly larger at 60% MVC than at 10% MVC in the right PM, the right IPL, and the primary somatosensory cortex (SI). These findings indicate that during motor imagery right fronto-parietal activity increases as the imagined contractile force level is intensified. The present finding that the right brain activity during motor imagery is clearly altered depending on the imagined force level suggests that it may be possible to decode intended force level during the motor imagery of patients or healthy subjects.

## Introduction

Motor imagery is defined as the mental execution of a movement without any overt movement. Neuroimaging studies using functional magnetic resonance imaging (fMRI) showed that motor imagery and execution share common neural substrates such as the supplemental motor area (SMA), premotor area (PM), primary motor cortex (Brodmann’s areas: BA 4), posterior parietal cortex (PPC), basal ganglia, and cerebellum (Decety et al., 1994; Lotze et al., 1999; Naito et al., 2002; Ehrsson et al., 2003; Hanakawa et al., 2003, 2008; Kuhtz-Buschbeck et al., 2003; Lacourse et al., 2005; Michelon et al., 2006; Higuchi et al., 2007; Imazu et al., 2007; Szameitat et al., 2007; Munzert et al., 2008; Chen et al., 2009; Guillot et al., 2009; Lorey et al., 2011; Mizuguchi et al., 2013a; Sharma and Baron, 2013). In addition, many studies report significant effects of motor imagery practice not only on motor skills but also on muscle strength (Feltz and Landers, 1983; Yue and Cole, 1992; Lotze and Halsband, 2006; Mizuguchi et al., 2012).

In voluntary motor execution, an accurate control of the appropriate force level is needed for precise motor performance. To date, numerous studies have investigated the relationship between brain activity and the level of contractile force. Single-cell recordings in animals indicate that there is a direct relationship between force level and the discharge rate of cortical neurons in BA 4 (Evarts, 1968), primary somatosensory cortex (SI; Wannier et al., 1991), and PM (Werner et al., 1991). In humans, an electrophysiological study by Perez and Cohen (2009) assessed corticospinal excitability by monitoring the magnitude of motor evoked potentials (MEPs) elicited by transcranial magnetic stimulation (TMS). They found that a graded modulation of corticospinal excitability during voluntary contractions. Studies utilizing neuroimaging have also found that brain activity in BA 4, SI, SMA, cingulate cortex, and cerebellum is correlated with contraction force level (Dettmers et al., 1995; Thickbroom et al., 1998; Dai et al., 2001; Ehrsson et al., 2001; Cramer et al., 2002). Taken together, the findings of the above studies indicate that valid measurements of the magnitude of contractile force can be assessed by monitoring brain activity via single cell discharge rate, MEPs, cerebral blood flow, and blood oxygen level dependent (BOLD) signal. However, during voluntary contractions brain activity must reflect not only the motor command but also the somatosensory afferent signals from the periphery. Therefore, it remains unclear as to whether brain activity reflects somatosensory input or motor command. Since there is no somatosensory input during motor imagery, brain activity during motor imagery would be expected to reflect only the motor command.

In a study evaluating whether corticospinal excitability during motor imagery is dependent upon imagined force level (Mizuguchi et al., 2013b), subjects practiced generating isometric forces of 10%, 30%, and 60% maximum voluntary contraction (MVC) before MEPs were recorded. Then, MEPs were measured during motor imagery of the same force generations. The MEPs amplitudes recorded in the agonist muscles of the 60% MVC condition were significantly greater than those of the 10% MVC condition. However, since the TMS study accessed only corticospinal excitability, it is still unclear whether activity in brain regions responsible for motor imagery other than the BA 4 correlate with the imagined force level. In the present study, we utilized fMRI to quantify BOLD signals and thereby establish a relationship between brain activity and imagined force level. We hypothesized that activity in motor regions such as the PM and SMA correlates with imagined force level.

## Materials and methods

### Subjects

Sixteen normal subjects (three females and thirteen males; mean age 22.9 ± 2.6 years) participated in this study. All of them were right-handed according to the Edinburgh Inventory (Oldfield, 1971). The subjects did not have a previous history of neurological or psychiatric disorders. Before the experiment, informed consent was obtained from all subjects. The study was approved by the Human Research Ethics committee of Waseda University.

### Procedure

The subjects performed three motor imagery conditions; (1) 10% MVC; (2) 30% MVC; and (3) 60% MVC. First, grip strength of the right hand was measured using an electronic hand dynamometer (EH101, Hata Sporting Goods Ind., Ltd., Osaka, Japan) outside the MRI room. The dynamometer was adjusted to best fit the grip of the subject’s right hand. Then, the subject was placed in a standing position and asked to squeeze the grip as hard as possible for 3 s without moving their arm. The subject was verbally encouraged to maximize the force. The subject performed this action twice with a 2-min rest between the trials. The mean value of the two trials was adopted as the subject’s MVC. After this determination, 10, 30, and 60% MVC of grip strength were calculated. Second, the subjects were instructed to match the grasping force at one of the three force levels for ten trials, with at least a 1-min rest between trials. After each trial, the subjects were given feedback regarding the difference between the performed and the target values. After ten trials of pre-training, the subjects moved to the MRI room immediately, where they performed one of the three conditions in the MRI room for 5 min 12 s. After the fMRI measurements, the subjects were moved outside of the MRI room, where they were again asked to match their force of grasping to the required % MVC for five trials with a hand dynamometer. They received no feedback about their performance. These procedures were repeated for the three different force levels. Thus, the subjects completed three different fMRI scans. The order of the three conditions of force level was randomized for each subject and counterbalanced across all subjects. Before performing the MVC measurement, the difference in motor imagery between the first person perspective and third person perspective (Stevens, 2005) was explained to the subjects. They were subsequently instructed to “imagine repeatedly grasping with the right hand using a first person perspective at your own pace in the fMRI experiment”. After each condition, we confirmed that the subjects conducted the instructed imagery. Approximately 5-min of rest was provided between conditions. In total, it took about 90 min for one subject to compete the entire experiment.

For the MRI scan, a run for each condition consisted of five alternate repetitions of the task and rest periods. The durations of the task and rest period were both 30 s. In the scan, the subjects were presented with a blue-filled or red-filled circle cue via a PC and projector system (VisuaStimDigital, Resonance Technology Co, USA). Each circle with a black background was presented with a non-magnetic goggle. When the blue cue was presented, the subjects were instructed to mentally reproduce the requested force without any muscle activation, with the right hand, at a natural and comfortable self-paced rhythm. In addition, they were asked to not change the pace during the experiment. When the red cue was shown, the subjects were asked to relax and not to image. The subjects were also asked to keep their muscles relaxed and not to think about anything throughout the entire procedure. Any communication between the experimenter and the subject was made through an intercom.

### Behavioral data analysis

The grasping forces produced by each subject were normalized with reference to the MVC of that particular subject. We then averaged the forces of the last of five trials in the pre fMRI and all of the five trials in the post fMRI period. Differences in the grasping force between the pre and post fMRI were evaluated with paired *t*-tests.

### fMRI data acquisition and analysis

All images were acquired using a 1.5 T MR scanner (Signa, General Electric, Wisc., USA). BOLD contrast functional images were acquired using T2*-weighted echo planar imaging (EPI) free induction decay (FID) sequences with the following parameters: TR 3000 ms, TE 50 ms, FOV 22 cm × 22 cm, flip angle 90°, slice thickness 5 mm and gap 1 mm. The orientation of the axial slices was parallel to the AC–PC line. For anatomical reference, T1-weighted images (TR 30 ms, TE 6 ms, FOV 24 cm × 24 cm, flip angle 90°, slice thickness 1 mm and no gap) were also obtained for each subject.

The first four volumes (12 s) of each fMRI session were discarded because of unstable magnetization. Raw data were analyzed utilizing Statistical Parametric Mapping (SPM8, Wellcome Department of Cognitive Neurology, London, UK) (Friston et al., 1994, 1995a,b) implemented in MATLAB (Mathworks, Sherborn, Massachusetts, USA). Realigned images were normalized to the standard space of the Montreal Neurological Institute brain (MNI brain). Then, smoothing was executed with an isotropic three-dimensional Gaussian filter with full-width at half-maximum (FWHM) of 8 mm. High-pass filters (128 s) were also applied and low frequency noise and global changes in the signals were removed. We confirmed that the subjects’ head movements were less than the size of one voxel.

Statistical analysis was performed on two levels. A first-level analysis was performed for each subject using a general linear model. We constructed a statistical parametric map of the *t*-statistic for the three contrasts, (1) 10% MVC vs. rest; (2) 30% MVC vs. rest; (3) 60% MVC vs. rest; (4) (60% MVC vs. rest) vs. (10% MVC vs. rest); (5) (60% MVC vs. rest) vs. (30% MVC vs. rest); and (6) (30% MVC vs. rest) vs. (10% MVC vs. rest). Subject-specific contrast images of the estimated parameter were used for a second-level analysis (random-effect model; Friston et al., 1999). The second-level analysis was performed to extend the inference from individual activation to the general population. One-sample *t* tests were used with a voxel-wise threshold of *p* < 0.001 (uncorrected) to generate the cluster images. Then, we set the threshold at *p* < 0.05 for the cluster level after correction by the false discovery rate (FDR) for the whole brain space. The locations of brain activity were transformed from MNI coordinates into Talairach standard brain coordinates (Talairach and Tournoux, 1988). If significant activation was found in the white matter, the result was excluded from description in the result section and tables.

We also calculated the BOLD signal changes that occurred during tasks in order to allow for the identification of activation peaks for each individual (Nakata et al., 2008). Eight regions were selected based on activation in the 60% MVC condition (Table 3), and each datum was collected from all subjects, using the “Plot” option in SPM8. The peak activities of three conditions in each region were analyzed by analyses of variance (ANOVAs) with repeated measures using as a within-subjects factor, condition (10% MVC, 30% MVC, and 60% MVC) (SPSS for windows version 21; IBM SPSS, Tokyo, Japan). For a repeated measures factor, it was tested whether Mauchly’s sphericity assumption was violated. In all cases, the sphericity was maintained, and it was not necessary to use the Greenhouse-Geisser correction. When significant effects were identified, *post hoc* analyses were determined by utilizing paired t-tests with the Bonferroni correction in each region. Statistical significance was set at *p* < 0.05 (*p* < 0.0166, uncorrected).

**Table 1:** **Activated regions in “10% MVC” > Rest**.

Region	Side	BA	Talairach Coordinates			*Z*-score
			*X*	*Y*	*Z*	
Frontal Lobe
Superior Frontal Gyrus	R	6	8	7	57	4.38
Medial Frontal Gyrus	L	6	−8	3	61	5.17
Middle Frontal Gyrus	L	10	−32	46	25	3.86
Precentral Gyrus	L	44	−61	8	11	4.37
Parietal Lobe
Postcentral Gyrus	L	40	−53	−30	51	3.78
Inferior Parietal Lobule	L	40	−55	−38	50	3.72
Temporal Lobe
Superior Temporal Gyrus	L	32	−61	−36	18	4.14
Limbic Lobe
Cingulate Gyrus	L	32	−4	17	38	4.12

**Table 2:** **Activated regions in “30% MVC” > Rest**.

Region	Side	BA	Talairach Coordinates			*Z*-score
			*X*	*Y*	*Z*
Frontal Lobe
Superior Frontal Gyrus	L	10	−36	51	16	3.18
Inferior Frontal Gyrus	L	9	−53	12	26	3.38
	L	44	−53	9	18	3.89
Medial Frontal Gyrus	L	6	−8	5	55	4.27
Middle Frontal Gyrus	L	10	−36	40	15	3.62
Precentral Gyrus	L	44	−50	8	5	4.02
Parietal Lobe
Postcentral Gyrus	L	2	−65	−22	29	4.24
Inferior Parietal Lobule	L	40	−53	−26	33	4.33
	R	40	59	−35	46	4.12
Supramarginal Gyrus	R	40	36	−37	30	4.01

**Table 3:** **Activated regions in “60% MVC” > Rest**.

Region	Side	BA	Talairach Coordinates			*Z*-score
			*X*	*Y*	*Z*
Frontal Lobe
Middle Frontal Gyrus	R	6	50	10	44	3.96
	R	10	38	53	5	4.29
	R	46	46	44	22	3.89
Parietal Lobe
Inferior Parietal Lobule	L	40	−51	−31	44	5.02
	R	40	46	−39	39	4.24
Postcentral Gyrus	R	2	55	−27	47	4.61
Limbic Lobe
Insula	R	13	44	8	−4	4.08
Cingulate Gyrus	R	32	16	19	29	4.78

## Results

### Behavioral data

**Figure 1:**
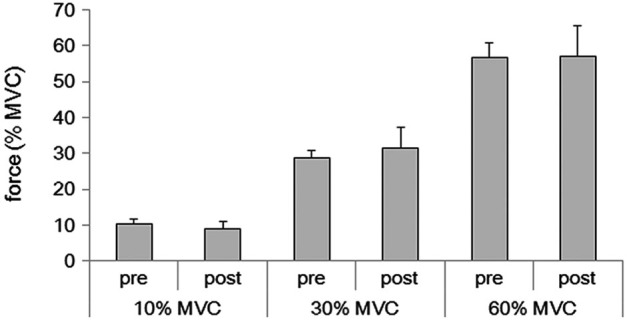
**Averages of grasping force in the pre and post fMRI for 10%, 30%, and 60% of the MVC for the 16 subjects**.

Subjects were able to reproduce each force level after the fMRI (10% MVC condition: 9.1 ± 1.9%, range 6.0–13.2%; 30% MVC condition: 31.5 ± 5.8%, range 20.5–40.8%; 60% MVC condition: 57.0 ± 8.4%, range 40.9–71.6%) (Figure 1). These values did not differ from the values obtained before the fMRI measurement (10% MVC condition: 10.2 ± 1.4%, range 7.3–13.0%; 30% MVC condition: 28.9 ± 1.8%, range 26.2–32.5%; 60% MVC condition: 56.8 ± 4.1%, range 47.2–64.7%) (*p* > 0.05, respectively).

### Imaging data

**Figure 2:**
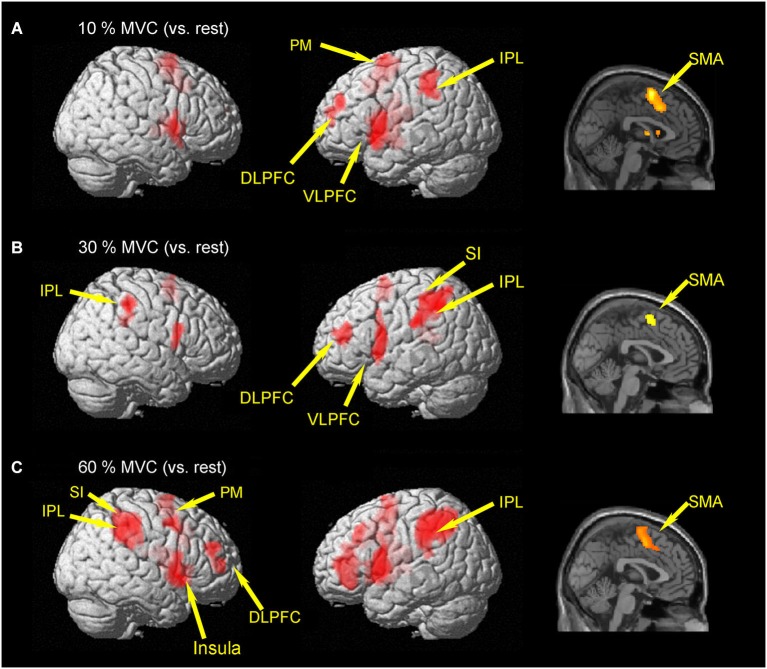
**Group activation map showing activated brain regions in each condition**. **(A)** 10% MVC vs. rest, **(B)** 30% MVC vs. rest, **(C)** 60% MVC vs. rest. Using the SPM8 template, areas showing an increase in BOLD-signal are superimposed on a 3D-rendered standard brain. DLPFC = dorsolateral prefrontal cortex; VLPFC = ventrolateral prefrontal cortex; IPL = inferior parietal lobule; PM = premotor area; SMA = supplementary motor area; SI = primary somatosensory cortex.

Brain activities related to the 10% MVC were located in the left dorsolateral prefrontal cortex (DLPFC) (BA 10), ventrolateral prefrontal cortec (VLPFC) (BA 44), PM (BA 6), SMA (BA 6), inferior parietal lobule (IPL) (BA 40), superior temporal gyrus (STG), and cingulate gyrus (BA 32). In the right hemisphere, activation was observed in the SMA (BA 6) (Figure 2A and Table 1).

Regions activated by the 30% MVC were located in the left DLPFC (BA 9 and 10), VLPFC (BA 44), superior frontal gyrus (SFG) (BA 10), SMA (BA 6), SI (BA 2), IPL (BA 40). Increased activity in the right hemisphere was located in the IPL (BA 40), and supramarginal gyrus (BA 40) (Figure 2B and Table 2).

Regions activated by the 60% MVC were located in the left IPL (BA 40). In the right hemisphere, significant activities were observed in the DLPFC (BA 10 and 46), PM (BA 6), IPL (BA 40), SI (BA 2), insula (BA 13), and cingulate gyrus (BA 32) (Figure 2C and Table 3). Since the cluster that included the right PM was extended to the left hemisphere, we analyzed sub-peak activities in the cluster. We also found significant activations in motor-related areas including the bilateral SMA, left DLPFC (BA 9), PM (BA6), insula (BA13), IFG (BA47).

We did not find significant differences in any voxels for the comparison of (60% MVC vs. rest) vs. (10% MVC vs. rest), (60% MVC vs. rest) vs. (30% MVC vs. rest), and (30% MVC vs. rest) vs. (10% MVC vs. rest).

### BOLD signal changes

**Figure 3:**
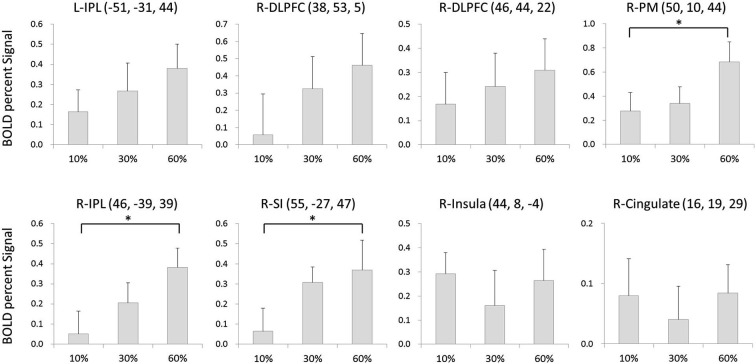
**The BOLD signal changes in 8 regions as compared among conditions**. There was a significant main effect of condition at right PM, right IPL and right SI. Error bars denote standard error (SE). * = *p* < 0.05 corrected.

The BOLD signal changes in eight regions were compared among conditions. There was a significant main effect of condition in the right PM (*F*_(2,30)_ = 6.216, *p* < 0.01), right IPL (*F*_(2,30)_ = 3.944, *p* < 0.05), and right SI (*F*_(2,30)_ = 4.946, *p* < 0.05). *Post-hoc* analysis showed that the activity was significantly larger at 60% of the MVC than at 10% MVC in the right PM (*p* < 0.05), larger for the 60% MVC than the 10% MVC in the right IPL (*p* < 0.05), and larger at 60% of the MVC than at 10% of the MVC in the right SI (*p* < 0.05). No statistically significant differences among conditions were found for other regions (Figure 3).

## Discussion

In the present study, we demonstrated that, for certain areas, brain activity during motor imagery was dependent upon imagined force level. We utilized force levels of 10%, 30%, and 60% of the MVC. We observed a common activation for the three force conditions in several brain regions. Such regions included the DLPFC, VLPFC, SMA, PM, insula, and IPL. This finding is consistent with previous neuroimaging studies examining motor imagery (Hanakawa et al., 2003, 2008; Lacourse et al., 2005; Lotze and Halsband, 2006; Imazu et al., 2007; Guillot et al., 2009; Mizuguchi et al., 2013a). Although we did not find significant differences in any voxels using a whole brain voxel-based analysis, the BOLD signal changes were significantly larger in the 60% MVC condition than they were in the 10% MVC conditions in the right PM, the right IPL, and SI. This discrepancy might be explained by the difference of the statistical power for the two situations.

The PM receives a strong input from the IPL (Rizzolatti and Luppino, 2001). Therefore, activity in the right PM and IPL is likely to be part of a fronto-parietal network. However, it is known that the function of the PM in motor execution differs from that of the IPL; that is, electrical stimulation of the PM triggers limb and mouth movements that are not consciously detected by the patients, whereas stimulation of the IPL produces the intention to move or the feeling that body parts have been moved, even in the absence of actual motor responses (Desmurget et al., 2009; Desmurget and Sirigu, 2009). Thus, it is likely that both the PM and IPL are linked to the imagination of force generation, but that these two regions function at different stages in the processing of motor imagery.

Studies utilizing fMRI and positron emission tomography (PET) during motor execution have provided evidence that activity in various regions such as BA 4, SI, PFC, SMA, PM, PPC, cingulate cortex, and cerebellum are correlated with the level of contracting force (Dettmers et al., 1995; Thickbroom et al., 1998; Ehrsson et al., 2000, 2001; Dai et al., 2001; Cramer et al., 2002). In addition, a non-human primate study demonstrated that neuronal activity in the PM contralateral to the active muscle is associated with the level of contractile force (Werner et al., 1991). In the current study, we showed that brain activity during motor imagery of force generation with the right hand was correlated with the imagined force level in the PM on the right (ipsilateral) side, and not on the left (Figure 3). One major difference between actual execution and motor imagery is the presence or absence of muscle contraction. That is, during muscle contraction, the contralateral BA 4 sends signals to motoneuron and the contralateral SI receives afferent feedback from muscle spindles and cutaneous receptors. The lack of a relationship between activity and imagined force level for the right hand, except for the right fronto-parietal region, might be due to the absence of afferent feedback from the periphery.

What are the functions of the right fronto-parietal region? A recent study utilizing diffusion tensor imaging demonstrated that the anatomical connection from the PM to other regions was different for the right and left hemispheres (van der Hoorn et al., 2014). According to this study, function of the right PM differs from that of the left PM. For example, the right PM has stronger connections to the occipito-parietal region of the opposite hemisphere, while the left PM has stronger connections to the prefrontal area and anterior parietal cortex. Indeed, previous studies suggest that the right fronto-parietal region play an important role in the integration of somatosensory input and motor command or in the induction of kinesthesia from somatosensory input (de Jong et al., 2002; Naito et al., 2005). Since the somatosensory cortices might receive efference copy from the motor cortices during motor imagery (Grush, 2004), we infer that the activity in the right fronto-parietal region seen in the present study is related to the amount of kinesthesia or efference copy during motor imagery. Other studies suggest that the right PM is related to motor awareness and sense of agency (Berti et al., 2005; Tsakiris et al., 2010). Since the subjects were required to imagine at higher effort level for the higher forces in the present study, activity in the right PM would be expected to also reflect a stronger motor command or greater effort to produce motor imagery. However, to clarify differences in the functions of the fronto-parietal region between the “right” and “left” hemispheres in more detail, future study will be need to perform the same analysis during motor imagery using the left hand at different force levels. A previous study has demonstrated that functional connectivity during motor imagery is different for kinesthetic motor imagery and visual imagery (Solodkin et al., 2004). In addition, functional connectivity during motor imagery is different between healthy subjects and stroke patients (Sharma et al., 2009). These results suggest that motor imagery ability and/or imagery modality affect the functional connectivity during motor imagery. Since the amount of brain activity was dependent upon imagined force levels, functional connectivity might also differ between higher and lower imagined force levels. In the future, we need to clarify whether functional connectivity during motor imagery is altered by different imagined force levels.

Recently, we reported that excitability of the corticospinal tract of the left hemisphere increased when the imagined force directed to the right hand increased (Mizuguchi et al., 2013b). Therefore, the left BA 4 would be expected to be associated with the imagined force level. Previous studies utilizing paired-pulse TMS demonstrated that activity of PM modulated excitability in the contralateral corticospinal tract via the transcallosal pathway (Mochizuki et al., 2004; Ni et al., 2009; Duque et al., 2012; Uehara et al., 2013). In addition, the right PM has an anatomical connection to the left precentral gyrus which includes a motor representation of the hand (van der Hoorn et al., 2014). Therefore, during motor imagery activity changes in the right PM might affect excitability in the left corticospinal tract via the transcallosal pathway. In the future, we need to clarify how the right PM or parietal region increased left corticospinal excitability during motor imagery with the right hand.

However, we did not see an activation of the BA 4 during motor imagery in the present study. While some researchers did find BA 4 activation during motor imagery (Porro et al., 1996; Lotze et al., 1999; Sharma et al., 2008; Chen et al., 2009; Guillot et al., 2009), others did not (Decety et al., 1994; Naito et al., 2002; Kuhtz-Buschbeck et al., 2003; Higuchi et al., 2007; Szameitat et al., 2007). This discrepancy might be associated with such factors as the degree of muscle activity, the type of tasks, and/or differneces between the subjects (Munzert et al., 2009). Kuhtz-Buschbeck et al. (2003) investigated brain activity and corticospinal excitability during motor imagery using fMRI and TMS. They found a significant enhancement of corticospinal excitability with TMS, but not significant activation in BA 4 when utilizing fMRI. These findings suggest the possibility that sensitivity for the detection of neural activation, especially in BA 4, was higher for TMS than for fMRI. Therefore, although we did not detect BA 4 activity in the present study, BA 4 might be active during motor imagery. Motor imagery ability is known to be correlated with the enhancement of corticospinal excitability during motor imagery (Williams et al., 2012). In the present study, we did not assess the motor imagery ability of each subject. Therefore, if we screened for motor imagery ability, we might have been able to detect BA 4 activity.

Another limitation of the present study was that we did not record an electromyogram for each muscle during motor imagery in the fMRI scan. Subjects might have contracted certain of their muscles during motor imagery. However, the lack of left BA 4 activation would indicate that actual muscle activity was minimal or absent during the motor imagery tasks. We believe that brain activity in the present study reflects motor imagery and not intended or unintended muscle activity.

In this study the relationship between neuronal activity and imagined force level was investigated. Our findings suggest that during motor imagery activity of the right fronto-parietal region increases as the imagined contractile force level with the right hand is intensified. Motor imagery can be utilized not only for rehabilitation and sports training but also as a mean to create a brain computer interface (e.g., Neuper et al., 2009). Thus, the main finding of this study–that right brain activity during motor imagery is clearly altered depending on the imagined force level–suggests that it may be possible to decode “intended force level” by monitoring activity in the right fronto-parietal region during the motor imagery of patients or healthy subjects.

## Conflict of interest statement

The authors declare that the research was conducted in the absence of any commercial or financial relationships that could be construed as a potential conflict of interest.

## References

[B1] Berti A. Bottini G. Gandola M. Pia L. Smania N. Stracciari A. (2005). Shared cortical anatomy for motor awareness and motor control. Science 15, 488–491 10.1126/science.1110625 16020740

[B2] Chen H. Yang Q. Liao W. Gong Q. Shen S. (2009). Evaluation of the effective connectivity of supplementary motor areas during motor imagery using Granger causality mapping. Neuroimage 47, 1844–1853 10.1016/j.neuroimage.2009.06.026 19540349

[B3] Cramer S. C. Weisskoff R. M. Schaechter J. D. Nelles G. Foley M. Finklestein S. P. (2002). Motor cortex activation is related to force of squeezing. Hum. Brain Mapp. 16, 197–205 10.1002/hbm.10040 12112762 PMC6871791

[B4] Dai T. H. Liu J. Z. Sahgal V. Brown R. W. Yue G. H. (2001). Relationship between muscle output and functional MRI-measured brain activation. Exp. Brain Res. 140, 290–300 10.1007/s002210100815 11681304

[B5] Decety J. Perani D. Jeannerod M. Bettinardl V. Tadary B. Woods R. (1994). Mapping motor representations with positron emission tomography. Nature 371, 600–602 10.1038/371600a0 7935791

[B6] de Jong B. M. Leenders K. L. Paans A. M. (2002). Right parieto-premotor activation related to limb-independent antiphase movement. Cereb. Cortex 12, 1213–1217 10.1093/cercor/12.11.1213 12379609

[B7] Desmurget M. Reilly K. T. Richard N. Szathmari A. Mottolese C. Sirigu A. (2009). Movement intention after parietal cortex stimulation in humans. Science 324, 811–813 10.1126/science.1169896 19423830

[B8] Desmurget M. Sirigu A. (2009). A parietal-premotor network for movement intention and motor awareness. Trends Cogn. Sci. 13, 411–419 10.1016/j.tics.2009.08.001 19748304

[B9] Dettmers C. Fink G. R. Lemon R. N. Stephan K. M. Passingham R. E. Silbersweig D. (1995). Relation between cerebral activity and force in the motor areas of the human brain. J. Neurophysiol. 74, 802–815 7472384 10.1152/jn.1995.74.2.802

[B10] Duque J. Labruna L. Verset S. Olivier E. Ivry R. B. (2012). Dissociating the role of prefrontal and premotor cortices in controlling inhibitory mechanisms during motor preparation. J. Neurosci. 32, 806–816 10.1523/JNEUROSCI.4299-12.2012 22262879 PMC3304578

[B11] Ehrsson H. H. Fagergren E. Forssberg H. (2001). Differential frontoparietal activation depending on force used in a precision grip task: an fMRI study. J. Neurophysiol. 85, 2613–2623 11387405 10.1152/jn.2001.85.6.2613

[B12] Ehrsson H. H. Fagergren A. Jonsson T. Westling G. Johansson R. S. Forssberg H. (2000). Cortical activity in precision- vs. power-grip tasks: an fMRI study. J. Neurophysiol. 83, 528–536 10634893 10.1152/jn.2000.83.1.528

[B13] Ehrsson H. H. Geyer S. Naito E. (2003). Imagery of voluntary movement of fingers, toes and tongue activates corresponding body-part-specific motor representations. J. Neurophysiol. 90, 3304–3316 10.1152/jn.01113.2002 14615433

[B14] Evarts E. V. (1968). Relation of pyramidal tract activity to force exerted during voluntary movement. J. Neurophysiol. 31, 14–27 4966614 10.1152/jn.1968.31.1.14

[B15] Feltz D. L. Landers D. M. (1983). The effects of mental practice on motor skill learning and performance: a meta-analysis. J. Sport Psychol. 5, 25–57

[B16] Friston K. J. Ashburner J. Frith C. D. Heather J. D. Frackowiak R. S. J. (1995a). Spatial registration and normalization of images. Hum. Brain Mapp. 3, 165–189 10.1002/hbm.460030303

[B17] Friston K. J. Holmes A. P. Worsley K. J. (1999). How many subjects constitute a study? Neuroimage 10, 1–5 10.1006/nimg.1999.0439 10385576

[B18] Friston K. J. Holmes A. P. Worsley K. J. Poline J.-P. Frith C. D. Frackowiak R. S. J. (1995b). Spatial parametric maps in functional imaging: a general linear approach. Hum. Brain Mapp. 2, 189–210 10.1002/hbm.460020402

[B19] Friston K. J. Jezzard P. Turner R. (1994). Analysis of functional MRI time-series. Hum. Brain Mapp. 1, 153–171 10.1002/hbm.460010207

[B21] Grush R. (2004). The emulation theory of representation: motor control, imagery and perception. Behav. Brain Sci. 27, 377–396; discussion 396–442 10.1017/s0140525x04000093 15736871

[B22] Guillot A. Collet C. Nguyen V. A. Malouin F. Richards C. Doyon J. (2009). Brain activity during visual versus kinesthetic imagery: an fMRI study. Hum. Brain Mapp. 30, 2157–2172 10.1002/hbm.20658 18819106 PMC6870928

[B24] Hanakawa T. Dimyan M. A. Hallett M. (2008). Motor planning, imagery and execution in the distributed motor network: a time-course study with functional MRI. Cereb. Cortex 18, 2775–2788 10.1093/cercor/bhn036 18359777 PMC2583155

[B25] Hanakawa T. Immisch I. Toma K. Dimyan M. A. Van Gelderen P. Hallett M. (2003). Functional properties of brain areas associated with motor execution and imagery. J. Neurophysiol. 89, 989–1002 10.1152/jn.00132.2002 12574475

[B26] Higuchi S. Imamizu H. Kawato M. (2007). Cerebellar activity evoked by common tool-use execution and imagery tasks: an fMRI study. Cortex 43, 350–358 10.1016/s0010-9452(08)70460-x 17533758

[B28] Imazu S. Sugio T. Tanaka S. Inui T. (2007). Differences between actual and imagined usage of chopsticks: an fMRI study. Cortex 43, 301–307 10.1016/s0010-9452(08)70456-8 17533754

[B29] Kuhtz-Buschbeck J. P. Mahnkopf C. Holzknecht C. Siebner H. Ulmer S. Jansen O. (2003). Effector-independent representations of simple and complex imagined finger movements: a combined fMRI and TMS study. Eur. J. Neurosci. 18, 3375–3387 10.1111/j.1460-9568.2003.03066.x 14686911

[B30] Lacourse M. G. Orr E. L. Cremer S. C. Cohen M. J. (2005). Brain activation during execution and motor imagery of novel and skilled sequential hand movements. Neuroimage 27, 505–519 10.1016/j.neuroimage.2005.04.025 16046149

[B32] Lorey B. Pilgramm S. Bischoff M. Stark R. Vaitl D. Kindermann S. (2011). Activation of the parieto-premotor networks is associated with vivid motor imagery—a parametric fMRI study. PLoS One 6:e20368 10.1371/journal.pone.0020368 21655298 PMC3105023

[B33] Lotze M. Halsband U. (2006). Motor imagery. J. Physiol. Paris 99, 386–395 10.1016/j.jphysparis.2006.03.012 16716573

[B34] Lotze M. Montoya P. Erb M. Hülsmann E. Flor H. Klose U. (1999). Activation of cortical and cerebellar motor areas during executed and imagined hand movement: an fMRI study. J. Cogn. Neurosci. 11, 491–501 10.1162/089892999563553 10511638

[B35] Michelon P. Vettel J. M. Zacks J. M. (2006). Lateral somatotopic organization during imagined and prepared movements. J. Neurophysiol. 95, 811–822 10.1152/jn.00488.2005 16207787

[B36] Mizuguchi N. Nakata H. Hayashi T. Sakamoto M. Muraoka T. Uchida Y. (2013a). Brain activity during motor imagery of an action with an object: a functional magnetic resonance imaging study. Neurosci. Res. 76, 150–155 10.1016/j.neures.2013.03.012 23562793

[B37] Mizuguchi N. Nakata H. Uchida Y. Kanosue K. (2012). Motor imagery and sport performance. J. Phys. Fit. Sports Med. 1, 103–111 10.7600/jpfsm.1.103

[B38] Mizuguchi N. Umehara I. Nakata H. Kanosue K. (2013b). Modulation of corticospinal excitability dependent upon imagined force level. Exp. Brain Res. 230, 243–249 10.1007/s00221-013-3649-3 23877227

[B39] Mochizuki H. Huang Y. Z. Rothwell J. C. (2004). Interhemispheric interaction between human dorsal premotor and contralateral primary motor cortex. J. Physiol. 561, 331–338 10.1113/jphysiol.2004.072843 15459244 PMC1665328

[B40] Munzert J. Lorey B. Zentgraf K. (2009). Cognitive motor processes: the role of motor imagery in the study of motor representations. Brain Res. Rev. 60, 306–326 10.1016/j.brainresrev.2008.12.024 19167426

[B41] Munzert J. Zentgraf K. Stark R. Vaitl D. (2008). Neural activation in cognitive motor processes: comparing motor imagery and observation of gymnastic movements. Exp. Brain Res. 188, 437–444 10.1007/s00221-008-1376-y 18425505

[B42] Naito E. Kochiyama T. Kitada R. Nakamura S. Matsumura M. Yonekura Y. (2002). Internally simulated movement sensations during motor imagery activate cortical motor areas and the cerebellum. J. Neurosci. 22, 3683–3691 11978844 10.1523/JNEUROSCI.22-09-03683.2002 PMC6758350

[B43] Naito E. Roland P. E. Grefkes C. Choi H. J. Eickhoff S. Geyer S. (2005). Dominance of the right hemisphere and role of area 2 in human kinesthesia. J. Neurophysiol. 93, 1020–1034 10.1152/jn.00637.2004 15385595

[B44] Nakata H. Sakamoto K. Ferretti A. Gianni Perrucci M. Del Gratta C. Kakigi R. (2008). Somato-motor inhibitory processing in humans: an event-related functional MRI study. Neuroimage 39, 1858–1866 10.1016/j.neuroimage.2007.10.041 18083602

[B45] Neuper C. Scherer R. Wriessnegger S. Pfurtscheller G. (2009). Motor imagery and action observation: modulation of sensorimotor brain rhythms during mental control of a brain-computer interface. Clin. Neurophysiol. 120, 239–247 10.1016/j.clinph.2008.11.015 19121977

[B46] Ni Z. Gunraj C. Nelson A. J. Yeh I. J. Castillo G. Hoque T. (2009). Two phases of interhemispheric inhibition between motor related cortical areas and the primary motor cortex in human. Cereb. Cortex 19, 1654–1665 10.1093/cercor/bhn201 19015374

[B47] Oldfield R. C. (1971). The assessment and analysis of handedness: the Edinburgh inventory. Neuropsychologia 9, 97–113 10.1016/0028-3932(71)90067-4 5146491

[B48] Perez M. A. Cohen L. G. (2009). Scaling of motor cortical excitability during unimanual force generation. Cortex 45, 1065–1071 10.1016/j.cortex.2008.12.006 19243741

[B49] Porro C. A. Francescato M. P. Cettolo V. Diamond M. E. Baraldi P. Zuiani C. (1996). Primary motor and sensory cortex activation during motor performance and motor imagery: a functional magnetic resonance imaging study. J. Neurosci. 16, 7688–7698 8922425 10.1523/JNEUROSCI.16-23-07688.1996 PMC6579073

[B50] Rizzolatti G. Luppino G. (2001). The cortical motor system. Neuron 31, 889–901 10.1016/s0896-6273(01)00423-8 11580891

[B51] Sharma N. Baron J. C. (2013). Does motor imagery share neural networks with executed movement: a multivariate fMRI analysis. Front. Hum. Neurosci. 7:564 10.3389/fnhum.2013.00564 24062666 PMC3771114

[B52] Sharma N. Baron J. C. Rowe J. B. (2009). Motor imagery after stroke: relating outcome to motor network connectivity. Ann. Neurol. 66, 604–616 10.1002/ana.21810 19938103 PMC3791355

[B53] Sharma N. Jones P. S. Carpenter T. A. Baron J. C. (2008). Mapping the involvement of BA 4a and 4p during motor imagery. Neuroimage 41, 92–99 10.1016/j.neuroimage.2008.02.009 18358742

[B54] Solodkin A. Hlustik P. Chen E. E. Small S. L. (2004). Fine modulation in network activation during motor execution and motor imagery. Cereb. Cortex 14, 1246–1255 10.1093/cercor/bhh086 15166100

[B55] Stevens J. A. (2005). Interference effects demonstrate distinct roles for visual and motor imagery during the mental representation of human action. Cognition 95, 329–350 10.1016/j.cognition.2004.02.008 15788162

[B56] Szameitat A. J. Shen S. Sterr A. (2007). Motor imagery of complex everyday movements. An fMRI study. Neuroimage 34, 702–713 10.1016/j.neuroimage.2006.09.033 17112742

[B57] Talairach J. Tournoux P. (1988). Co-Planar Stereotaxic Atlas of the Human Brain. New York: Thieme

[B58] Thickbroom G. W. Phillips B. A. Morris I. Byrnes M. L. Mastaglia F. L. (1998). Isometric force-related activity in sensorimotor cortex measured with functional MRI. Exp. Brain Res. 121, 59–64 10.1007/s002210050437 9698191

[B59] Tsakiris M. Longo M. R. Haggard P. (2010). Having a body versus moving your body: neural signatures of agency and body-ownership. Neuropsychologia 48, 2740–2749 10.1016/j.neuropsychologia.2010.05.021 20510255

[B60] Uehara K. Morishita T. Kubota S. Funase K. (2013). Neural mechanisms underlying the changes in ipsilateral primary motor cortex excitability during unilateral rhythmic muscle contraction. Behav. Brain Res. 240, 33–45 10.1016/j.bbr.2012.10.053 23174210

[B61] van der Hoorn A. Potgieser A. R. de Jong B. M. (2014). Transcallosal connection patterns of opposite dorsal premotor regions support a lateralized specialization for action and perception. Eur. J. Neurosci. 40, 2980–2986 10.1111/ejn.12656 24945328

[B62] Wannier T. M. Maier M. A. Hepp-Reymond M. C. (1991). Contrasting properties of monkey somatosensory and motor cortex neurons activated during the control of force in precision grip. J. Neurophysiol. 65, 572–589 2051196 10.1152/jn.1991.65.3.572

[B63] Werner W. Bauswein E. Fromm C. (1991). Static firing rates of premotor and primary motor cortical neurons associated with torque and joint position. Exp. Brain Res. 86, 293–302 10.1007/bf00228952 1756804

[B64] Williams J. Pearce A. J. Loporto M. Morris T. Holmes P. (2012). The relationship between corticospinal excitability during motor imagery and motor imagery ability. Behav. Brain Res. 226, 369–375 10.1016/j.bbr.2011.09.014 21939692

[B65] Yue G. Cole K. J. (1992). Strength increases from the motor program: comparison of training with maximal voluntary and imagined muscle contractions. J. Neurophysiol. 67, 1114–1123 1597701 10.1152/jn.1992.67.5.1114

